# How antipsychotics work in schizophrenia: a primer on mechanisms

**DOI:** 10.1017/S1092852924002244

**Published:** 2024-12-02

**Authors:** Jonathan M. Meyer

**Affiliations:** Voluntary Clinical Professor of Psychiatry, University of California, San Diego, CA, USA

**Keywords:** schizophrenia, antipsychotic, mechanism, negative symptoms, cognitive dysfunction, dopamine, clozapine, xanomeline

## Abstract

Antipsychotics effective for schizophrenia approved prior to 2024 shared the common mechanism of postsynaptic dopamine D_2_ receptor antagonism or partial agonism. Positive psychosis symptoms correlate with excessive presynaptic dopamine turnover and release, yet this postsynaptic mechanism improved positive symptoms only in some patients, and with concomitant risk for off-target motor and endocrine adverse effects; moreover, these agents showed no benefit for negative symptoms and cognitive dysfunction. The sole exception was data supporting cariprazine’s superiority to risperidone for negative symptoms. The muscarinic M_1_/M_4_ agonist xanomeline was approved in September 2024 and represents the first of a new antipsychotic class. This novel mechanism improves positive symptoms by reducing presynaptic dopamine release. Xanomeline also lacks any D_2_ receptor affinity and is not associated with motor or endocrine side effects. Of importance, xanomeline treated patients with higher baseline levels of cognitive dysfunction in clinical trials data saw cognitive improvement, a finding likely related to stimulation of muscarinic M_1_ receptors. Treatment resistance is seen in one-third of schizophrenia patients. These individuals do not have dopamine dysfunction underlying their positive symptoms, and therefore show limited response to antipsychotics that target dopamine neurotransmission. Clozapine remains the only medication with proven efficacy for resistant schizophrenia, and with unique benefits for persistent impulsive aggression and suicidality. New molecules are being studied to address the array of positive, negative and cognitive symptoms of schizophrenia; however, until their approval, clinicians must be familiar with currently available agents and be adept at prescribing clozapine.

## Introduction

Schizophrenia spectrum disorders are characterized by core central nervous system (CNS) domains: positive symptoms (hallucinations, delusions, disorganized speech/behavior); negative symptoms (apathy/avolition, diminished expression); and cognitive dysfunction (deficits in working memory, processing speed, executive function).^1^ Positive symptoms are necessary to establish the diagnosis, but patients vary considerably in both the presentation of those symptoms, and the extent and severity of negative symptoms and cognitive deficits. Other associated features of schizophrenia include high rates of substance use disorders,^2^ persistent depressive symptoms,^3^ and twofold higher rates of aggression,^4^ with the latter being a product of inadequately controlled positive symptoms or of impulsivity not motivated by psychosis.^5^ A distinct neurobiological substrate underlies each of these symptom clusters, and multiple neurotransmitters are implicated in the dysfunction of relevant circuits, particularly dopamine, glutamate, acetylcholine (ACh), and serotonin.^6^ ^-^^9^

Given the complex neurobiology of schizophrenia, and the reality that each individual has their own distinct clinical presentation, no antipsychotic effectively remediates the totality of the three primary symptom domains, with cognitive dysfunction and negative symptoms exhibiting limited benefit from most agents.^10^ ^,^^11^ This limitation is likely rooted in the common mechanism of action for most antipsychotics approved prior to 2024: dopamine D_2_ receptor blockade. This mechanism is responsible for any improvements in positive symptoms, but has limited independent benefit for negative and cognitive symptoms. D_2_ receptor blockade is also inadequate to manage positive symptoms in roughly one-third of patients (ie those with treatment resistant schizophrenia [TRS]).^12^ ^-^^14^ Although D_2_ receptor binding has been the model for most antipsychotics, there are two agents whose primary antipsychotic mechanism lies outside of this domain: clozapine, and the first of a new class of medication that lacks any D_2_ receptor affinity (xanomeline) but instead works by stimulating a subset of muscarinic cholinergic receptors.^15^ ^,^^16^ Clozapine binds weakly to the D_2_ receptor, but it clearly possesses other mechanisms. To date clozapine remains the only medication with proven efficacy in TRS, namely those with inadequate positive symptom response to D_2_ binding antipsychotics.^12^ ^,^^17^ Moreover, clozapine exhibits other unique clinical properties in patients with schizophrenia, including reduction in suicidal behavior and impulsive aggression, and alleviation of psychogenic polydipsia (ie excessive water drinking related to poorly controlled psychosis).^4^ ^,^^17^ Clozapine’s mechanisms of action remain incompletely understood despite US approval for TRS over 35 years ago on September 26, 1989, although one hypothesis is discussed below in the section on TRS.^18^ Importantly, despite advances in the neuropharmacology of schizophrenia, there is no compelling evidence that any other antipsychotic, including the new muscarinic receptor activators, are effective substitutes for clozapine in TRS, or for schizophrenia patients with persistent aggression or suicidality not responsive to D_2_ receptor modulating agents.^17^

## Positive symptoms

**Figure 1.**
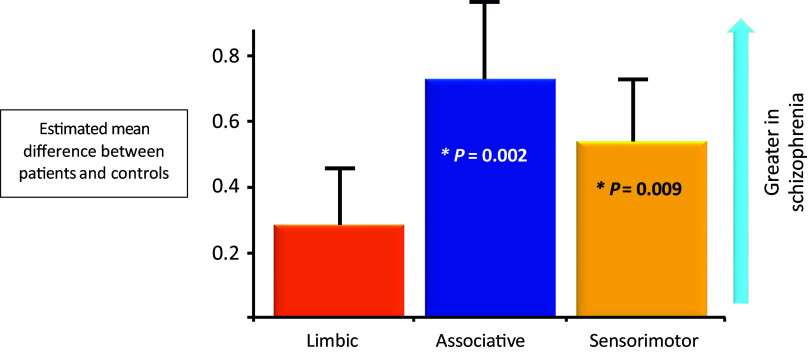
Imaging findings note presynaptic dopamine dysfunction (excessive turnover and release) in the associative and adjacent sensorimotor areas of the striatum for patients with schizophrenia when compared to control subjects.^13^

Although clozapine’s efficacy profile has not been replicated, D_2_ receptor binding antipsychotics and muscarinic antipsychotic agents share a core property: reduction in dopamine neurotransmission. How this is achieved varies greatly between the two classes of medication, but that difference is best understood in the context of the dopamine dysfunction inherent to positive symptoms.^13^ Human imaging studies demonstrate that the positive symptoms in schizophrenia patients *who are not treatment resistant* are associated with excess presynaptic production of dopamine in the associative striatum (Figure 1). ^13^ ^,^^18^ This understanding was not present in the early 1950s when two competing antipsychotic mechanisms became commercially available: depletion of dopamine from presynaptic neurons by reserpine,^19^ or blockade of postsynaptic dopamine receptors by chlorpromazine.^20^ The first widely imitated antipsychotic, chlorpromazine (Thorazine®), was initially synthesized in 1950 as an improvement on an earlier compound promethazine (Phenergan®). The goal was to develop a more potent medication to induce a nonnarcotic state of “artificial hibernation” and thereby ease anesthetic induction and post-surgery recovery.^20^ The connection with dopamine was only later elucidated by Arvid Carlsson, a discovery that garnered Carlsson the Nobel Prize in Physiology or Medicine in 2000.^20^ Carlsson’s insight was to connect the finding that motor symptoms of Parkinson’s disease were related to loss of dopamine producing neurons, and the observation that medications effective for positive psychotic symptoms (eg chlorpromazine or reserpine) were associated with a reversible form of drug-induced parkinsonism (DIP). From those facts he deduced in 1963 that antipsychotic medications must be blocking dopamine receptors, or, in the case of reserpine, act by depleting dopamine from presynaptic stores.^21^ Carlsson’s inductive leap was that the underlying pathophysiology of positive symptoms must somehow relate to excessive dopamine in a specific brain circuit, thereby formulating the dopamine hypothesis of schizophrenia. Animal models characterized the dopamine tracts involved in positive symptoms, and modern human imaging studies confirmed the association of positive symptoms with excessive dopamine turnover in the associative striatum and adjacent portions of the sensorimotor striatum.^13^

Although positive symptoms are a presynaptic problem of dopamine overproduction and release, the presynaptic mechanism inherent to reserpine (blockade of the vesicular monoamine transporter type 2 [VMAT2]) was abandoned as the basis for future antipsychotics by the early 1960s after trials of another VMAT2 inhibitor tetrabenazine.^22^ Tetrabenazine shared reserpine’s core mechanism and lacked reserpine’s effects on blood pressure, but it proved no more effective than reserpine or chlorpromazine, and was often associated with akathisia (restlessness) and DIP at the doses needed to control psychosis.^23^ ^,^^24^ With VMAT2 inhibition reaching a dead end, Carlsson’s discovery that chlorpromazine’s impact on positive psychotic symptoms rested in dopamine receptor blockade facilitated development of compounds that shared its mechanism (D_2_ receptor antagonism), but without chlorpromazine’s risk for sedation, orthostasis, and anticholinergic adverse effects (eg dry mouth, memory impairment, constipation).^25^ Subsequent generations of D_2_ acting antipsychotics were later developed that possessed lower risk for DIP, tardive dyskinesia (TD), and other movement disorders related to D_2_ receptor blockade^14^; however, *when used in equivalent dosages*, all antipsychotics were comparably effective in non-TRS patients (Table 1).^26^

**Table 1.** Antipsychotics Listed Alphabetically and by Primary Mechanism for Positive Symptom Reduction

First generation (D_2_ receptor antagonists)	Second generation (D_2_ receptor antagonists)	Second generation (D_2_ receptor partial agonists)	Muscarinic M_1_/M_4_ receptor stimulating agents
Chlorpromazine Fluphenazine Haloperidol Perphenazine	Asenapine *Clozapine* ^a^ Iloperidone Lumateperone Lurasidone Olanzapine Paliperidone Quetiapine Risperidone Ziprasidone	Aripiprazole Brexpiprazole Cariprazine	Xanomeline-trospium

a Clozapine is the only effective medication for treatment resistant schizophrenia

**Figure 2.**
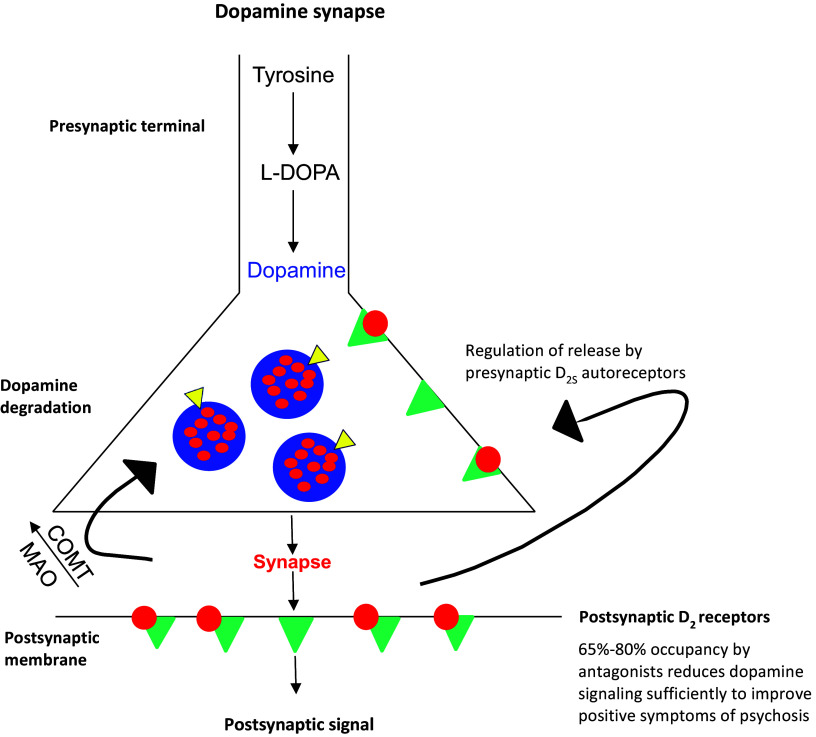
How dopamine D_2_ receptor binding antipsychotics work at dopamine synapses.^14^ *Scheme:* Dopamine—red dots; blue circles—presynaptic vesicles containing dopamine; yellow triangles—vesicular monoamine transporter type 2 (VMAT2); dopamine D_2_ receptors—green triangles; *Abbreviations:* MAO: monoamine oxidase; COMT: catechol *O*-methyltransferase. Legend: *Dopamine is produced in the presynaptic neuron by conversion from tyrosine to L-dopa and then to dopamine. Dopamine is inserted into presynaptic vesicles by VMAT2, and is released into the synapse upon neuronal stimulation. Excess synaptic dopamine is broken down via the enzymes COMT or MAO. D_2_ antagonist antipsychotics bind to both presynaptic and postsynaptic D_2_ receptors. Blocking dopamine on the presynaptic autoreceptor further disinhibits presynaptic dopamine release. To improve positive symptoms, D_2_ antagonist antipsychotics must block 65%–80% of postsynaptic receptors. The three dopamine partial agonist antipsychotics require 80%–100% postsynaptic receptor occupancy for effective antipsychotic activity.*

Following the demise of presynaptic acting VMAT2 inhibitors, the mechanism of action for every antipsychotic approved through 2023 involved blockade of postsynaptic dopamine D_2_ receptors. As illustrated in Figure 2, these agents did not address the presynaptic basis of positive symptoms, but managed this problem by interfering with dopamine binding at postsynaptic receptors.^14^ These antipsychotics were nonselective, and acted at D_2_ receptors throughout the CNS and in the periphery yielding several unfortunate consequences. At the level of the dopamine synapse, D_2_ antagonists blocked postsynaptic D_2_ receptors but also blocked the shorter variant D_2S_ receptors present on presynaptic neurons.^14^ As these presynaptic D_2S_ receptors are inhibitory, blocking dopamine’s activity further disinhibits presynaptic dopamine release. The level of receptor occupancy required for D_2_ antagonists to overcome this effect was not understood when antipsychotics first became available, and efficacy was established for dosage ranges that managed positive symptoms while minimizing as much as possible motor adverse effects.^14^ Only in the late 1980s did imaging studies find that at least 65% postsynaptic D_2_ receptor occupancy was associated with positive symptom reduction, while >80% receptor occupancy was associated with higher rates of motor adverse effects resulting from D_2_ blockade in the dorsal striatum (referred to as extrapyramidal side effects in the older literature): DIP, akathisia, and TD. The proverbial “sweet spot” for D_2_ receptor occupancy was thus in the range of 65%–80%, but with significant interindividual heterogeneity noted in the correlation between occupancy, response, and tolerability.^27^ First-generation antipsychotics (FGAs) had significantly higher rates of D_2_-related motor effects compared to second-generation antipsychotics (SGAs), as the latter possessed an inherent mechanism to mitigate this risk in the form of serotonin 2A (5HT_2A_) receptor antagonism.^28^ ^,^^29^ Three dopamine partial agonist antipsychotics (DPAs) were developed (aripiprazole, brexpiprazole, cariprazine) that also have lower risk of motor side effects than FGAs due to their weak intrinsic dopaminergic activity.^14^ Because these agents weakly stimulate postsynaptic D_2_ receptors, imaging studies noted that DPAs became effective for positive symptoms at 80%–100% D_2_ receptor occupancy. This level of D_2_ occupancy would pose significant tolerability problems for antagonist antipsychotics, but the intrinsic dopamine activity of the DPAs results in relatively low rates of DIP and akathisia.^14^

Two other unfortunate consequences of D_2_ receptor antagonism are sexual dysfunction from blockade of D_2_ receptors in the hypothalamic–pituitary axis (HPA), and glucose dysregulation.^14^ As dopamine inhibits prolactin release from the HPA, D_2_ receptor blockade can induce hyperprolactinemia of sufficient severity to lower sex hormone levels resulting in menstrual irregularities, gynecomastia or galactorrhea, decreased libido, and bone density loss.^14^ ^,^^30^ Blockade of D_2_ receptors on insulin secreting pancreatic β-cells and in glucose sensing hypothalamic cells impairs glycemic control, thereby putting patients at risk for metabolic syndrome and diabetes mellitus.^31^

Xanomeline is a muscarinic M_1_ and M_4_ receptor agonist initially developed to improve cognition in Alzheimer’s disease, but was surprisingly found to exert antipsychotic properties in those patients despite being devoid of any D_2_ receptor binding.^32^ Subsequent animal research discovered that the dopamine neurons associated with positive symptoms receive cholinergic and glutamatergic stimulatory input, and that stimulation of M_1_ and M_4_ receptors lessen the extent of this input. Cholinergic input to the relevant dopamine tracts originates from a midbrain structure, the laterodorsal tegmental nucleus (LDT).^8^ ^,^^16^ LDT neurons possess an abundance of inhibitory M_4_ autoreceptors—therefore, any agent which stimulates M_4_ receptors will decrease LDT ACh output, with the net result being decreased ACh stimulation of presynaptic dopamine outflow and a reduction in positive symptoms.^16^ ^,^^33^ Although there is cholinergic stimulation of dopaminergic neurons in motor areas of the striatum, this cholinergic pathway (the pedunculopontine nucleus) is primarily controlled by activity at M_2_ autoreceptors. Muscarinic M_4_ receptor stimulating molecules (agonists or positive allosteric modulators) thus work presynaptically to reduce positive symptoms, yet they do so without D_2_ receptor binding, and they act selectively, sparing motor areas from effects on dopamine neurotransmission.^33^

Muscarinic M_1_ receptor activation also acts selectively to decrease presynaptic dopamine output, but the antipsychotic effect arises via modulation of the stimulatory glutamate signal that originates in the prefrontal cortex (PFC).^16^ Glutamate signaling from the PFC is decreased by stimulating M_1_ receptors on inhibitory GABA-ergic interneurons in the PFC. Increased activity of these GABA-ergic interneurons acts as a brake on glutamate outflow, with the net result seen as less glutamate stimulated dopamine release and less positive symptoms.^16^ Stimulation of M_1_ receptors is associated with gastrointestinal adverse effects, so xanomeline was subsequently combined with trospium, an anticholinergic medication that does not appreciably cross the blood brain barrier and thus mitigates the procholinergic adverse effects of peripheral M_1_ agonism without interfering with xanomeline’s CNS mechanism.^15^ ^,^^34^ ^,^^35^ Use of anticholinergics with extensive CNS penetration (eg benztropine, diphenhydramine) is strongly discouraged when treating patients with schizophrenia due to their deleterious cognitive effects,^36^ but there is now another reason to eschew these agents: they will interfere with the action of muscarinic receptor stimulating antipsychotics.^37^ On the basis of three positive trials, xanomeline-trospium received FDA approval on September 26, 2024, exactly 35 years after that for clozapine. Unlike the example of clozapine, xanomeline’s mechanism is better understood and forms the basis for a new class of muscarinic receptor stimulating agents currently undergoing clinical trials for schizophrenia and other psychotic disorders.^16^ The obvious advantage lies in the fact that their selective presynaptic mechanism reduces dopamine overactivity, but without the motor or endocrine adverse effects seen with D_2_ receptor binding antipsychotics.^16^ Moreover, the presynaptic mechanism provided by muscarinic receptor stimulating antipsychotics can work cooperatively with postsynaptic D_2_ receptor blockade to lessen the impact of excessive dopamine signaling.^38^ For that reason, clinicians and researchers who work in the field of schizophrenia are eagerly awaiting data from a randomized study of xanomeline-trospium or placebo added adjunctively to D_2_ acting antipsychotics. This trial (A Study to Assess Efficacy and Safety of Adjunctive KarXT in Subjects With Inadequately Controlled Symptoms of Schizophrenia; NCT05145413) is due to report data in 2025.

### Clozapine for TRS or schizophrenia with persistent aggression

One-third of patients living with schizophrenia are treatment resistant, and thus realize little to no positive symptom reduction from D_2_ receptor modulation.^12^ Imaging studies indicate that TRS is associated with relatively normal striatal dopamine synthesis, not the excessive presynaptic dopamine turnover and release typically associated with positive symptoms, thus explaining why these patients derive limited benefit from D_2_ receptor blockade.^6^ At least 40% of those with TRS will respond to clozapine, while response to other antipsychotics, even at high dosages, is typically <5%.^39^ ^,^^40^ When imaged with proton magnetic resonance spectroscopy, response to clozapine in TRS patients is associated with reduction of the glutamate signal in the caudate, but the exact mechanism by which clozapine exerts this effect is not sufficiently characterized to the extent it has been replicated by other molecules.^18^ Given the high prevalence of TRS, use of clozapine becomes critical to competency restoration when persistent positive symptom severity impedes adjudication.^41^

Clozapine possesses another unique benefit—an effect on aggression that is independent of its impact on psychosis symptoms.^4^ Multiple factors, especially substance misuse, underlie behaviors that bring patients with psychotic disorders into contact with the criminal justice system.^42^ Poorly controlled positive symptoms are an important contributor to elevated violence risk in patients living with schizophrenia, so aggression remains a core target of antipsychotic therapy.^43^ However, it should be noted that the most common form of interpersonal violence in forensic inpatient populations is not psychotically driven—it is impulsive aggression related to inadequate control over response to provocative stimuli.^5^ ^,^^42^ A detailed analysis of 839 assaults among chronically aggressive state hospital patients noted that only 17% were motivated by psychosis (or mania), while 54% were impulsive, and the remaining 29% were planned or predatory in nature.^44^ When persistent aggression or violence in schizophrenia patients is due to undertreated psychosis, the usual treatment algorithm is followed to address positive symptoms.^5^ When aggressive behaviors in that patient population are impulsive, the most strongly evidence-based pharmacological intervention is clozapine.^4^ ^,^^5^ A 2024 review of clozapine’s anti-aggression effects found that this property existed for impulsive aggression in patients whose positive symptoms were adequately controlled.^4^ One of the most compelling pieces of evidence was the findings from a prospective, double-blind trial of clozapine, olanzapine and haloperidol in persistently aggressive male state hospital patients with modest levels of psychotic symptoms.^45^ ^,^^46^ That study found clozapine superior to the other medications for acts of aggression, with no differences between the three medications on psychosis symptoms; moreover, clozapine’s anti-aggression effect was particularly evident in patients with greater baseline levels of cognitive dysfunction.^45^ ^,^^46^

Clozapine’s treatment-related adverse effects and hematological monitoring requirements are a burden for patients with schizophrenia, and often dissuade clinicians from its use despite the absence of evidence-based options for TRS or persistent impulsive aggression.^17^ As decades of research have failed to uncover the mix of receptor activities that result in its unparalleled effectiveness, it is incumbent that clinicians working with forensic populations develop expertise in prescribing clozapine.^47^ ^,^^48^ As noted in the literature, the failure to prescribe clozapine to TRS patients or schizophrenia patients with persistent aggression is deemed to be below the standard of care as it deprives incarcerated patients of the fundamental right to effective treatment.^49^ ^,^^50^

## Negative symptoms

The differential diagnosis of negative symptoms includes those which are inherent to the diagnosis of schizophrenia (ie primary) or those due to other causes such as depression, anxiety, or medication induced adverse effects.^51^ It should be noted that antipsychotic trials of acutely exacerbated adult schizophrenia patients find negative symptom improvement, but the extent of this improvement is highly correlated with positive symptom reduction, a phenomenon known as pseudospecificity.^52^ ^-^^54^ Stable, modestly symptomatic patients with persistent moderate/severe primary negative symptoms achieve limited negative symptom benefit from most antipsychotics.^51^ Although the complex neurobiology of negative symptoms has thwarted attempts at developing approved agents, they remain an important treatment target given the high prevalence and associated disability. It is worth noting that the DPA cariprazine demonstrated comparative benefit on negative symptoms versus the D_2_ receptor antagonist SGA risperidone in a 26-week randomized, double-blind, controlled trial (n = 461), with a modest effect size of 0.31.^55^ Among the three DPAs, cariprazine possesses the highest affinity for the D_3_ receptor, and it is the only one in this antipsychotic class effective as monotherapy for bipolar depression.^56^ ^,^^57^ Although patients with moderate or severe depressive symptoms were excluded from that trial, it is unclear if cariprazine’s negative symptom impact lies outside of its antidepressant mechanisms, or is an epiphenomenon of these receptor activities.^57^

## Cognitive dysfunction

Cognitive impairment associated with schizophrenia (CIAS) is a common and disabling feature of the disorder clinically recognized for over a century. It was the presence of prominent cognitive disturbance that led Emil Kraepelin to arrive at the term *dementia praecox* (premature dementia) for this psychotic disorder.^14^ CIAS has two aspects in common with negative symptoms: (1) there can be secondary causes of cognitive dysfunction that must be addressed (eg benzodiazepines, CNS acting anticholinergics, sedatives) and (2) the complex neurobiology of CIAS and the heterogeneity of symptoms has hindered progress in producing effective agents.^9^ Nonetheless, ongoing studies continue to focus on this disabling feature of schizophrenia, with medications in clinical trials that work by stimulating *N*-methyl-D-aspartate (NMDA) glutamate receptors.^58^ ^,^^59^ The underlying hypothesis driving development of these agents is that hypofunction of NMDA receptors residing on PFC GABA-ergic interneurons contributes to CIAS.^60^ The NMDA receptor possesses a binding site for glutamate, and a co-agonist site that binds either glycine or D-serine.^61^ The leading candidates stimulate the co-agonist site by one of two strategies: inhibiting glycine reuptake to increase synaptic levels of glycine (iclepertin), or inhibiting the metabolism of D-serine thereby increasing its synaptic levels (luvadaxistat).^58^ ^,^^59^ Sadly, luvadaxistat failed to meet its primary endpoints in a second phase 2 study and further research was abandoned by the manufacturer.^62^

The discovery of xanomeline’s antipsychotic properties not only opened new avenues for positive symptom control, it also refocused attention on one aspect of schizophrenia neurobiology that relates to CIAS, and which may be improved by xanomeline’s M_1_ receptor agonism: low muscarinic M_1_ receptor expression.^63^ ^,^^64^ Although initially noted in postmortem specimens,^64^ subsequent imaging studies found modestly decreased M_1_ receptor density in unmedicated antipsychotic naïve schizophrenia patients compared to age-matched peers without schizophrenia.^65^ Further research noted that 25% of schizophrenia patients have ≥75% decreased M_1_ receptor density, a subgroup referred to as having the muscarinic receptor deficit subgroup (MRDS).^64^ Schizophrenia patients with MRDS show widespread decreases in cortical M_1_ receptors, altered patterns of M_1_ receptor gene promoter methylation, and lower levels of muscarinic M_1_ receptor mRNA compared to controls.^65^ Notably, non-MRDS patients with schizophrenia do not differ in these measures from control individuals. Not surprisingly, lower levels of muscarinic M_1_ receptor expression are associated with poorer performance in verbal learning and memory and more severe negative symptoms in medication free psychotic patients.^65^

Since any pool of schizophrenia patients possessing severe cognitive deficits would be enriched with those having MRDS, the hypothesis that xanomeline’s M_1_ receptor stimulation might improve CIAS was explored as a secondary outcome measure in clinical trials.^66^ Neuroimaging for low M_1_ expression was not possible, but analysis of the double-blind phase 2b study found differential cognitive benefits from xanomeline stratified by level of impairment.^66^ As seen in Table 2, the cognitive impact of xanomeline devolved only to the subgroup with clinically significant cognitive impairment (defined as a baseline composite cognitive battery score more than one standard deviation below the normative mean).^66^ This finding of cognitive benefit in cognitively impaired patients, presumably from xanomeline’s M_1_ activity, aligns with the concept that more severe forms of CIAS are associated with MRDS, while schizophrenia patients with limited cognitive dysfunction likely have CNS M_1_ expression and activity closer to the norm. Importantly, the association of xanomeline treatment with improved cognitive function in impaired patients was replicated in exploratory analyses from the two phase three studies.^67^ These positive results represent the first breakthrough in CIAS treatment, findings that should be particularly noteworthy to the field of forensic psychiatry. For schizophrenia patients who are not treatment resistant but whose level of cognitive dysfunction remains an impediment to competency restoration, xanomeline may offer potential hope to address CIAS symptoms that interfere with mastery of court material and effective interaction with attorneys and other court personnel.

**Table 2.** Xanomeline-trospium treatment effect on cognitive performance by baseline impairment in a double-blind, placebo controlled phase 2b trial^a^ ^66^

	LS mean change from baseline at day 35			
	Treatment arm	Estimate (SE)	*p* Value	Cohen’s *d*
**Minimally impaired**	KarXT (n = 34)	−0.18 (0.13)	0.19	0.22
	Placebo (n = 65)	−0.22 (0.15)	0.15	0.28
	KarXT vs. placebo	0.04 (0.16)	0.79	0.05
**Impaired**	KarXT (n = 23)	0.57 (0.19)	**0.01**	**0.61**
	Placebo (n = 37)	0.07 (0.13)	0.59	0.09
	KarXT vs. placebo	0.50 (0.22)	**0.03**	**0.50**

Least squares (LS) means and *p* values are derived from post hoc analysis of covariance (ANCOVA) models, with covariates of site, gender, age, and baseline performance.

a For this exploratory analysis, individuals with a high degree of test subdomain intraindividual variability were removed as this is typically reflective of noncompliance with test procedures or otherwise invalid data.

## Conclusion

Despite the disability resulting from negative symptoms and cognitive dysfunction, the clinical effect of antipsychotics was historically dependent on D_2_ receptor blockade and the benefit largely confined to positive symptom reduction. Yet 2024 saw a revolution in positive symptom treatment, providing clinicians two means to manage the consequences of presynaptic dopamine overactivity: blocking dopamine from binding to postsynaptic dopamine D_2_ receptors, or reducing presynaptic dopamine release by stimulation of muscarinic M_1_ and M_4_ receptors. Importantly, muscarinic receptor stimulation not only avoids the motor and endocrine adverse effects of nonselective D_2_ blockade, clinical trials of xanomeline-trospium noted cognitive benefits among patients with significant levels of cognitive dysfunction. The promise of cognitive improvement had not been realized previously and hopefully diminishes the level of clinical nihilism when confronted with this important problem. Despite these advances, clozapine remains the only effective medication for resistant schizophrenia or schizophrenia patients with persistent impulsive aggression, and its complex interplay of pharmacological activities has defied replication in molecules with improved tolerability. Given the absence of other effective options for TRS or persistent impulsive aggression, all clinicians who treat patients with schizophrenia must be adept at using clozapine—it is the standard of care.

## Data Availability

Not applicable (this is a review paper).

## References

[1] Marder SR, Cannon TD. Schizophrenia. N Engl J Med. 2019;381(18):1753–1761. doi:10.1056/NEJMra1808803 31665579

[2] Siddiqui S, Mehta D, Coles A, Selby P, Solmi M, Castle D. Psychosocial interventions for individuals with comorbid psychosis and substance use disorders: systematic review and meta-analysis of randomized studies Schizophr Bull. 2024;doi:10.1093/schbul/sbae101 38938221

[3] Ceskova E. Pharmacological strategies for the management of comorbid depression and schizophrenia. Expert Opin Pharmacother. 2020;21(4):459–465. doi:10.1080/14656566.2020.1717466 31983254

[4] Faden J, Citrome L. A systematic review of clozapine for aggression and violence in patients with schizophrenia or schizoaffective disorder. Schizophr Res. 2024;268:265–281. doi:10.1016/j.schres.2023.11.008 38290941

[5] Meyer JM, Cummings MA, Proctor G, Stahl SM. Psychopharmacology of persistent violence and aggression. Psychiatr Clin North Am. 2016;39(4):541–556. 27836150 10.1016/j.psc.2016.07.012

[6] Egerton A, Murphy A, Donocik J, et al. Dopamine and glutamate in antipsychotic-responsive compared with antipsychotic-nonresponsive psychosis: a multicenter positron emission tomography and magnetic resonance spectroscopy study (STRATA). Schizophr Bull. 2021;47(2):505–516. doi:10.1093/schbul/sbaa128 32910150 PMC7965076

[7] Iasevoli F, Avagliano C, D’Ambrosio L, et al. Dopamine dynamics and neurobiology of non-response to antipsychotics, relevance for treatment resistant schizophrenia: a systematic review and critical appraisal. Biomedicines. 2023;11(3)doi:10.3390/biomedicines11030895 PMC10046109 36979877

[8] McCutcheon RA, Weber LAE, Nour MM, Cragg SJ, McGuire PM. Psychosis as a disorder of muscarinic signalling: psychopathology and pharmacology. Lancet Psychiatry. 2024;11(7):554–565. doi:10.1016/S2215-0366(24)00100-7 38795721

[9] Howes OD, Bukala BR, Beck K. Schizophrenia: from neurochemistry to circuits, symptoms and treatments. Nat Rev Neurol. 2024;20(1):22–35. doi:10.1038/s41582-023-00904-0 38110704

[10] Kantrowitz JT, Correll CU, Jain R, Cutler AJ. New developments in the treatment of schizophrenia: an expert roundtable. Int J Neuropsychopharmacol. 2023;26(5):322–330. doi:10.1093/ijnp/pyad011 36932673 PMC10229849

[11] Howes OD, Dawkins E, Lobo MC, Kaar SJ, Beck K. New drug treatments for schizophrenia: a review of approaches to target circuit dysfunction. Biol Psychiatry. 2024;doi:10.1016/j.biopsych.2024.05.014 38815885

[12] Howes OD, McCutcheon R, Agid O, et al. Treatment-resistant schizophrenia: Treatment Response and Resistance in Psychosis (TRRIP) working group consensus guidelines on diagnosis and terminology. Am J Psychiatry. 2017;174(3):216–229. doi:10.1176/appi.ajp.2016.16050503 27919182 PMC6231547

[13] McCutcheon RA, Abi-Dargham A, Howes OD. Schizophrenia, dopamine and the striatum: from biology to symptoms. Trends Neurosci. 2019;42(3):205–220. doi:10.1016/j.tins.2018.12.004 30621912 PMC6401206

[14] Meyer JM. Ch 19—Pharmacotherapy of Psychosis and Mania. In: Brunton LL, ed. Goodman & Gilman’s The Pharmacological Basis of Therapeutics, 14th ed. McGraw-Hill; 2022:357–384:chap 19.

[15] Kaul I, Sawchak S, Walling DP, et al. Efficacy and safety of xanomeline-trospium chloride in schizophrenia: a randomized clinical trial. JAMA Psychiatry. 2024;doi:10.1001/jamapsychiatry.2024.0785 PMC11063924 38691387

[16] Paul SM, Yohn SE, Brannan SK, Neugebauer NM, Breier A. Muscarinic receptor activators as novel treatments for schizophrenia. Biol Psychiatry. 2024;doi:10.1016/j.biopsych.2024.03.014 38537670

[17] Meyer JM, Stahl SM. The Clozapine Handbook—Stahl’s Handbooks. Cambridge University Press; 2019:317.

[18] McQueen G, Sendt KV, Gillespie A, et al. Changes in brain glutamate on switching to clozapine in treatment-resistant schizophrenia. Schizophr Bull. 2021;47(3):662–671. doi:10.1093/schbul/sbaa156 33398325 PMC8084451

[19] Bleuler M, Stoll WA. Clinical use of reserpine in psychiatry: comparison with chlorpromazine. Ann N Y Acad Sci. 1955;61(1):167–73. doi:10.1111/j.1749-6632.1955.tb42463.x 14377284

[20] Lopez-Munoz F, Alamo C, Cuenca E, Shen WW, Clervoy P, Rubio G. History of the discovery and clinical introduction of chlorpromazine. Ann Clin Psychiatry 2005;17(3):113–35. doi:10.1080/10401230591002002 16433053

[21] Carlsson A, Lindqvist M. Effect of chlorpromazine or haloperidol on formation of 3-methoxytyramine and normetanephrine in mouse brain. Acta Pharmacol Toxicol (Copenh). 1963;20:140–4. doi:10.1111/j.1600-0773.1963.tb01730.x 14060771

[22] Quinn GP, Shore PA, Brodie BB. Biochemical and pharmacological studies of RO 1-9569 (tetrabenazine), a nonindole tranquilizing agent with reserpine-like effects. J Pharmacol Exp Ther. 1959;127(1):103–109. 14435563

[23] Smith ME. Clinical comparison of tetrabenazine (Ro 1-9569), reserpine and placebo in chronic schizophrenics. Dis Nerv Syst. 1960;21(3)Suppl:120–123. 13832091

[24] Ashcroft GW, Macdougall EJ, Barker PA. A comparison of tetrabenazine and chlorpromazine in chronic schizophrenia. J Ment Sci. 1961;107:287–93. doi:10.1192/bjp.107.447.287 13684728

[25] Janssen PA. The evolution of the butyrophenones, haloperidol and trifluperidol, from meperidine-like 4-phenylpiperidines. Int Rev Neurobiol. 1965;8:221–263. doi:10.1016/s0074-7742(08)60759-x 5321473

[26] Huhn M, Nikolakopoulou A, Schneider-Thoma J, et al. Comparative efficacy and tolerability of 32 oral antipsychotics for the acute treatment of adults with multi-episode schizophrenia: a systematic review and network meta-analysis. The Lancet. 2019;394(10202):939–951. doi:10.1016/s0140-6736(19)31135-3 PMC6891890 31303314

[27] Kapur S, Zipursky R, Jones C, Remington G, Houle S. Relationship between dopamine D(2) occupancy, clinical response, and side effects: a double-blind PET study of first-episode schizophrenia. Am J Psychiatry. 2000;157(4):514–20. 10739409 10.1176/appi.ajp.157.4.514

[28] Bubser M, Backstrom JR, Sanders-Bush E, Roth BL, Deutch AY. Distribution of serotonin 5-HT(2A) receptors in afferents of the rat striatum. Synapse. 2001;39(4):297–304. doi:10.1002/1098-2396(20010315)39:4<297::Aid-syn1012>3.0.Co;2-q 11169779

[29] Navailles S, De Deurwaerdère P. Presynaptic control of serotonin on striatal dopamine function. Psychopharmacology (Berl). 2011;213(2–3):213–42. doi:10.1007/s00213-010-2029-y 20953589

[30] Dibonaventura M, Gabriel S, Dupclay L, Gupta S, Kim E. A patient perspective of the impact of medication side effects on adherence: results of a cross-sectional nationwide survey of patients with schizophrenia. BMC Psychiatry. 2012;12:20. doi:10.1186/1471-244x-12-20 22433036 PMC3342101

[31] Castellani LN, Pereira S, Kowalchuk C, et al. Antipsychotics impair regulation of glucose metabolism by central glucose. Mol Psychiatry. 2022;27(11):4741–4753. doi:10.1038/s41380-022-01798-y 36241692

[32] Bodick NC, Offen WW, Levey AI, et al. Effects of xanomeline, a selective muscarinic receptor agonist, on cognitive function and behavioral symptoms in Alzheimer disease. Arch Neurol. 1997;54(4):465–73. doi:10.1001/archneur.1997.00550160091022 9109749

[33] Yohn SE, Weiden PW, Felder CC, Stahl SM. Muscarinic acetylcholine receptors for psychotic disorders: bench-side to clinic. Trends Pharmacol Sci. 2022;43(12):1098–1112. doi:10.1016/j.tips.2022.09.006 36273943

[34] Brannan SK, Sawchak S, Miller AC, Lieberman JA, Paul SM, Breier A. Muscarinic cholinergic receptor agonist and peripheral antagonist for schizophrenia. N Engl J Med. 2021;384(8):717–726. doi:10.1056/NEJMoa2017015 33626254 PMC7610870

[35] Kaul I, Sawchak S, Correll CU, et al. Efficacy and safety of the muscarinic receptor agonist KarXT (xanomeline-trospium) in schizophrenia (EMERGENT-2) in the USA: results from a randomised, double-blind, placebo-controlled, flexible-dose phase 3 trial. Lancet. 2024;403(10422):160–170. doi:10.1016/s0140-6736(23)02190-6 38104575

[36] Joshi YB, Thomas ML, Braff DL, et al. Anticholinergic medication burden-associated cognitive impairment in schizophrenia. Am J Psychiatry. 2021;178(9):838–847. doi:10.1176/appi.ajp.2020.20081212 33985348 PMC8440496

[37] Barak S, Weiner I. The M₁/M₄ preferring agonist xanomeline reverses amphetamine-, MK801- and scopolamine-induced abnormalities of latent inhibition: putative efficacy against positive, negative and cognitive symptoms in schizophrenia. Int J Neuropsychopharmacol. 2011;14(9):1233–46. doi:10.1017/s1461145710001549 21211109

[38] Kinon BJ, Leucht S, Tamminga C, Breier A, Marcus R, Paul SM. Rationale for adjunctive treatment targeting multiple mechanisms in schizophrenia. J Clin Psychiatry. 2024;85(3)doi:10.4088/JCP.23nr15240 39196873

[39] Kane J, Honigfeld G, Singer J, Meltzer H. Clozapine for the treatment-resistant schizophrenic. A double-blind comparison with chlorpromazine. Arch Gen Psychiatry. 1988;45(9):789–96. 3046553 10.1001/archpsyc.1988.01800330013001

[40] Siskind D, Siskind V, Kisely S. Clozapine response rates among people with treatment-resistant schizophrenia: data from a systematic review and meta-analysis. Can J Psychiatry. 2017;62(11):772–777. doi:10.1177/0706743717718167 28655284 PMC5697625

[41] Singh A, Delgado D, Ventura MI, Schwartz E, Williams J, Meyer JM. Clozapine use and forensic outcomes in psychiatric inpatients deemed incompetent to stand trial. J Am Acad Psychiatry Law. 2022;50(3):427–433. doi:10.29158/jaapl.210123-21 35798392

[42] Lambe S, Cooper K, Fazel S, Freeman D. Psychological framework to understand interpersonal violence by forensic patients with psychosis. Br J Psychiatry. 2024;224(2):47–54. doi:10.1192/bjp.2023.132 37861077 PMC10807973

[43] Whiting D, Gulati G, Geddes JR, Dean K, Fazel S. Violence in schizophrenia: triangulating the evidence on perpetration risk. World Psychiatry. 2024;23(1):158–160. doi:10.1002/wps.21171 38214634 PMC10785986

[44] Quanbeck CD, McDermott BE, Lam J, Eisenstark H, Sokolov G, Scott CL. Categorization of aggressive acts committed by chronically assaultive state hospital patients. Psychiatr Serv. 2007;58(4):521–8. doi:10.1176/appi.ps.58.4.521 17412855

[45] Krakowski MI, Czobor P, Citrome L, Bark N, Cooper TB. Atypical antipsychotic agents in the treatment of violent patients with schizophrenia and schizoaffective disorder. Arch Gen Psychiatry. 2006;63(6):622–629. 16754835 10.1001/archpsyc.63.6.622

[46] Krakowski MI, Czobor P. Executive function predicts response to antiaggression treatment in schizophrenia: a randomized controlled trial. J Clin Psychiatry. 2012;73(1):74–80. doi:10.4088/JCP.11m07238 22152404

[47] Tibrewal P, Nair PC, Gregory KJ, Langmead CJ, Chan SKW, Bastiampillai T. Does clozapine treat antipsychotic-induced behavioural supersensitivity through glutamate modulation within the striatum? Mol Psychiatry. 2023;28(5):1839–1842. doi:10.1038/s41380-023-02026-x 36932159 PMC10575773

[48] Naguy A, Alhazeem H. Clozapine prescripers-dogmatic or pragmatic? Psychopharmacol Bull. 2024;54(2):46–50. 38601835 10.64719/pb.4490 PMC11003257

[49] Zarzar TR, Williams JB, Pruette ME, Sheitman BB. A legal right to clozapine therapy for incarcerated individuals with treatment-resistant schizophrenia. Psychiatr Serv. 2021;doi:10.1176/appi.ps.202000845 33593106

[50] Zarzar TR. Clozapine proficiency as a milestone in psychiatric training. JAMA Psychiatry. 2024;81(7):639–640. doi:10.1001/jamapsychiatry.2024.0702 38691351

[51] Correll CU, Schooler NR. Negative symptoms in schizophrenia: a review and clinical guide for recognition, assessment, and treatment. Neuropsychiatr Dis Treat. 2020;16:519–534. doi:10.2147/NDT.S225643 32110026 PMC7041437

[52] Hopkins SC, Ogirala A, Loebel A, Koblan KS. Understanding antipsychotic drug treatment effects: a novel method to reduce pseudospecificity of the positive and negative syndrome scale (PANSS) factors. Innov Clin Neurosci. 2017;14(11–12):54–58. 29410937 PMC5788251

[53] Hopkins SC, Ogirala A, Loebel A, Koblan KS. Transformed PANSS factors intended to reduce pseudospecificity among symptom domains and enhance understanding of symptom change in antipsychotic-treated patients with schizophrenia. Schizophr Bull. 2018;44(3):593–602. doi:10.1093/schbul/sbx101 28981857 PMC5890480

[54] Hopkins SC, Ogirala A, Loebel A, Koblan KS. Characterization of specific and distinct patient types in clinical trials of acute schizophrenia using an uncorrelated PANSS score matrix transform (UPSM). Psychiatry Res. 2020;294:113569. doi:10.1016/j.psychres.2020.113569 33223272

[55] Nemeth G, Laszlovszky I, Czobor P, et al. Cariprazine versus risperidone monotherapy for treatment of predominant negative symptoms in patients with schizophrenia: a randomised, double-blind, controlled trial. Lancet. 2017;389(10074):1103–1113. doi:10.1016/S0140-6736(17)30060-0 28185672

[56] Girgis RR, Slifstein M, D’Souza D, et al. Preferential binding to dopamine D3 over D2 receptors by cariprazine in patients with schizophrenia using PET with the D3/D2 receptor ligand [(11)C]-(+)-PHNO. Psychopharmacology (Berl). 2016;233(19–20):3503–12. doi:10.1007/s00213-016-4382-y 27525990 PMC5035321

[57] Stahl SM. Mechanism of action of cariprazine. CNS Spectr. 2016;21(2):123–7. doi:10.1017/s1092852916000043 26956157

[58] Rosenbrock H, Desch M, Wunderlich G. Development of the novel GlyT1 inhibitor, iclepertin (BI 425809), for the treatment of cognitive impairment associated with schizophrenia. Eur Arch Psychiatry Clin Neurosci. 2023;273(7):1557–1566. doi:10.1007/s00406-023-01576-z 36971864 PMC10465677

[59] Murthy V, Hanson E, DeMartinis N, et al. INTERACT: a randomized phase 2 study of the DAAO inhibitor luvadaxistat in adults with schizophrenia. Schizophr Res. 2024;270:249–257. doi:10.1016/j.schres.2024.06.017 38943928

[60] Tamminga CA. The neurobiology of cognition in schizophrenia. J Clin Psychiatry. 2006;67 Suppl 9:9–13; discussion 36–42. 16965183

[61] Peng A, Chai J, Wu H, et al. New therapeutic targets and drugs for schizophrenia beyond dopamine D2 receptor antagonists. Neuropsychiatr Dis Treat. 2024;20:607–620. doi:10.2147/ndt.S455279 38525480 PMC10961082

[62] Neurocrine Biosciences Inc. Neurocrine Biosciences provides update on ERUDITE™ phase 2 data for luvadaxistat in adults with cognitive impairment associated with schizophrenia. https://www.neurocrine.com/our-company/news-and-media/news/neurocrine-biosciences-provides-update-on-erudite-phase-2-data-for-luvadaxistat-in-adults-with-cognitive-impairment-associated-with-schizophrenia/. Accessed September 12, 2024.

[63] Gibbons AS, Scarr E, Boer S, et al. Widespread decreases in cortical muscarinic receptors in a subset of people with schizophrenia. Int J Neuropsychopharmacol. 2013;16(1):37–46. doi:10.1017/s1461145712000028 22338582

[64] Dean B, Haroutunian V, Scarr E. Lower levels of cortical [3H]pirenzepine binding to postmortem tissue defines a sub-group of older people with schizophrenia with less severe cognitive deficits. Schizophr Res. 2023;255:274–282. doi:10.1016/j.schres.2023.03.035 37079947

[65] Dean B, Scarr E. Muscarinic M1 and M4 receptors: hypothesis driven drug development for schizophrenia. Psychiatry Res. 2020;288:112989. doi:10.1016/j.psychres.2020.112989 32315882

[66] Sauder C, Allen LA, Baker E, Miller AC, Paul SM, Brannan SK. Effectiveness of KarXT (xanomeline-trospium) for cognitive impairment in schizophrenia: post hoc analyses from a randomised, double-blind, placebo-controlled phase 2 study. Transl Psychiatry. 2022;12(1):491. doi:10.1038/s41398-022-02254-9 36414626 PMC9681874

[67] Horan W, Sauder C, Harvey PD, Ramsay IS, Paul SM, Brannan SK. The impact of KarXT on cognitive impairment in acute schizophrenia: replication in pooled data from phase 3 trials. *Poster 88 presented at the 2024 Annnual Congress of the Schizophrenia International Research Society, 3–7 April 2024, Florence, Italy*; 2024.

